# Supplementary material for: An inventory of human night-biting mosquitoes and their bionomics in Sumba, Indonesia
Source: PLoS Negl Trop Dis. 2022 Mar 21;16(3):e0010316. doi: 10.1371/journal.pntd.0010316 (PMC8970493; doi:10.1371/journal.pntd.0010316)
Supplement: Attachment — Submitted filename: PNTD-D-21-01348_ReviewerComments.docx [file pntd.0010316.s002.docx]

General summary

This study gives a detailed account of the human biting mosquitoes found in one specific region of Indonesia, based on 3 years of sampling and over 73,000 mosquitoes collected. The study is well designed and provides some basic – important – information about seasonality, biting times, and biting location (in vs. outdoor) for all genera of mosquitoes collected during HLCs. The study also provides a catalogue of species found within these genera (in the study site), with more detail given for *Anopheles* based on the parent study. The manuscript is generally well organized and well written (with some exceptions noted below). I have separated my remaining comments as either major (critical for a clear understanding or presentation of the study) or minor (less critical, but could improve manuscript in some way).

Major comments

- Lines 69-70: The Author Summary states, “Morphological and molecular analyses of 73,507 collected mosquito specimens revealed…”. However, this contradicts with the abstract, which states, “…with a subset being morphologically and molecularly identified”. Please correct this.
- Author Summary: The writing in this section is less polished than the rest of the manuscript, and some of the points raised (potential risks of HLC; discrepancy between morphological ID and molecular ID) feel incomplete in the current form. I would suggest giving this section a thorough review and update.
- Line 151: what is meant by “subset”? Were HLCs done at houses in all 12 clusters every two weeks? If not, how many clusters were in each subset?
- Lines 158-164:
  - Please provide more details about the random selection of samples. A flow chart may be helpful to provide a clear understanding. This could be a supplemental figure.
  - For sequencing, it seems that samples were first stratified by morphological species ID, cluster and time period. Is that correct? If so, I think would be helpful to state that explicitly. Also, please include the target number of specimens per strata. If that target number was different, e.g. by species, this is where a flow chart may be especially helpful.
  - For random selection of specimens for morphological identification, was a similar stratification process used?
- Line 193: please include in the results some kind of statement about the total number of person-nights sampled.
- Table 1:
  - Please give more detail about the % identity given for each molecular species. Did all specimens ID’d as a given species have the same % identity?
  - Please include the number of specimens ID’d as each molecular species (can be in parentheses after name), as well as the number of specimens ID’d as each morphological species within a given row.
  - Please clarify what is meant by “not including actual species”. For the first 3 genera (at least), the actual species seems to be listed many times (e.g. *Cx. quinquefasciatus, Ae. vexans*)
- Lines 301-303: please clarify whether this “first-time evaluation..” applies globally, to Indonesia, to Sumba (or to some other level), to give a better understanding of the uniqueness of this rich dataset.

Minor comments

- Line 52: I think “Anopheline” should not be capitalized. Please change to either Anophelinae or anopheline. (also line 54)
- Line 57: “Anopheles” should be italicized.
- Lines 70-71: Please check the punctuation after “*Culex”*
- Line 89: the subfamilies typically end in -inae, so Toxorhynchites should be Toxorhynchitinae.
- Lines 108-110: I assume this is meant to say something like, “Characterizing species-specific traits…”.
- Line 126: Is the ecological analysis mentioned here meant to be part of species identification *per se*? Or rather, is it that we can only accurately characterize species-specific ecology using data from accurately identified mosquito specimens?
- Line 139 and Figure 2: should start date for rainfall data collection be June 2015? If not, where did data in Figure 2 come from prior to July 2016? Also, line 248 says, “Weather stations recorded rainfall and temperature throughout the study period”.
- Lines 140-142: please edit this section for clarity. Also, should this include temperature? (temperature is mentioned on line 180 but omitted in lines 140-142).
- Figure 1: please add the number of houses at each cluster/star in the legend text.
- Line 194: Should starting month be May 2015 as indicated in Abstract, Author Summary, and Methods? (Or else update the other 3 sections?)
- Line 220: Suggested change to “There were seven *Aedes* taxa identified molecularly”, or something other than species, given that five remained unidentified. Similar suggestion on lines 227 and 231.
- Lines 253-254: in the text, correlation with rainfall is given for Culicinae, and stated as a positive correlation, but then the p-value is given as p<0.48, which would not be significant. Please check this section for accuracy.
- Table 2: some of the decimals are written as commas but should be changed to periods.
- Lines 254-255: “Peaks between rainfall highs are were associated with agricultural irrigation.” Was this formally assessed in some way? If not, I would suggest to leave that for the discussion section.
- Lines 268-271: These lines could be removed given that the information is all contained in the figure.
- Lines 328-331: Clarify whether these species are known/documented JEV vectors.
- Line 361: Clarify in this first sentence that the HBRs are specifically for evening/night, and not daytime biting.
- References should be checked. For example:
  - Ref. 3 is a study specifically about vectors of JEV in Indonesia, and so seems inappropriate as a citation for global mosquito taxa. A more appropriate reference would be, e.g., <https://doi.org/10.1016/B978-012510451-7/50014-1>
  - Ref. 6 is the World Malaria Report, but on line 96 is used as a reference for filarial worms and their vectors.
