# Supplementary material for: An inventory of human night-biting mosquitoes and their bionomics in Sumba, Indonesia
Source: PLoS Negl Trop Dis. 2022 Mar 21;16(3):e0010316. doi: 10.1371/journal.pntd.0010316 (PMC8970493; doi:10.1371/journal.pntd.0010316)
Supplement: Attachment — Submitted filename: Response to Reviewers_020222.docx [file pntd.0010316.s003.docx]

**Response to Reviewers**

We thank the reviewers for their constructive comments and suggestions

Below are the point-by-point responses

Please note that several edits and improvements have been made throughout the manuscript.

**Major comments**

1. ***Question****: Lines 69-70: The Author Summary states, “Morphological and molecular analyses of 73,507 collected mosquito specimens revealed…”. However, this contradicts with the abstract, which states, “…with a subset being morphologically and molecularly identified”. Please correct this.*

**Authors Answer:** Thank you. We have clarified this statement as follows:

“The collection and analysis of 73,507 mosquito specimens revealed the presence of various species of *Anopheles*, *Aedes*, *Culex, Armigeres* and *Mansonia,* that potentially transmit several diseases such as malaria, filaria, dengue and many other mosquitoes borne viral diseases.”

2. ***Question:*** *Author Summary: The writing in this section is less polished than the rest of the manuscript, and some of the points raised (potential risks of HLC; discrepancy between morphological ID and molecular ID) feel incomplete in the current form. I would suggest giving this section a thorough review and update*.

**Authors Answer:**

Thank you. The Author Summary has been rewritten to better represent the overall study results in lay language.

3. ***Question:*** *Line 151: what is meant by “subset”? Were HLCs done at houses in all 12 clusters every two weeks? If not, how many clusters were in each subset?*

**Authors Answer:** Thank you for this clarifying question. The term subset refers to the subset of the parent study clusters that were utilized for entomological collections. The parent study had 24 clusters - of which only (a subset) 12 were utilized for HLCs. The term ‘subset’ has been removed from the text for clarity.

4. ***Question:*** *Lines 158-164: Please provide more details about the random selection of samples. A flow chart may be helpful to provide a clear understanding. This could be a supplemental figure.*

**Authors Answer:** Samples were stratified by morphological species, cluster, location, and time of capture, and a random subset (representing at least 10% of each morphologically identified species) spanning all strata were selected for sequencing. For example, if there were 1000 specimens of a specific species identified, at least 10% (100 specimens) were randomly sampled to represent all sites, location of capture (inside and outside), and time of capture (across the night as well as over the course of the entire study).

5. ***Question.*** For sequencing, it seems that samples were first stratified by morphological species ID, cluster and time period. Is that correct? If so, I think would be helpful to state that explicitly. Also, please include the target number of specimens per strata. If that target number was different, e.g. by species, this is where a flow chart may be especially helpful.

**Authors Answer:** Thank you. You are correct. There was no target number of specimens per strata as the number of each species present differed both spatially and temporally. Note that the number of specimens sequenced were also limited by the available budget. A best effort was made to maximize the number of specimens sequenced to represent all strata. We have reworded the methods section to be more clear (see previous question):

**‘**Samples were stratified by morphological species, cluster, location, and time of capture, and a random subset (representing at least 10% of each morphologically identified species) spanning all strata were selected for…’

***Question:*** *For random selection of specimens for morphological identification, was a similar stratification process used?*

**Authors Answer:** morphological identification was carried out for all Anopheline specimens. Morphological identification were not conducted for all non-Anopheline specimens. Primary morphological identification for non-Anophelines were carried out at the genera level and a random subset (limited by time and budget) were identified morphologically to species (similar to that with the molecular identifications).

6. ***Question:*** *Line 193: please include in the results some kind of statement about the total number of person-nights sampled*

**Authors Answer:** Thank you. There were a total of 52 nights of HLCs, in 12 clusters, and in 4 houses each (inside and outside) which totals 2,496 indoor and 2,496 outdoor person nights (total of 4992 person nights). This has been added in to the methods section.

7. ***Question:*** *Table 1:*

*Please give more detail about the % identity given for each molecular species. Did all specimens ID’d as a given species have the same % identity?*

**Authors Answer:** The % identity used for each molecular species is exactly the same as that used in the references sited. In short, each contig required sequences to have at least 98% identity for CO1, and 96% for ITS2 for inclusion. Each contig was examined for insertions and/or deletions with iterative assembly (using higher and lower identity thresholds) to include or parse out sequences. Consequentially, sequences with the same species identity determined may have had up to a 2% difference for CO1 and 4% with ITS2 – without any higher matches in the dataset.

***Question:*** *Please include the number of specimens ID’d as each molecular species (can be in parentheses after name), as well as the number of specimens ID’d as each morphological species within a given row*

**Authors Answer:** Thank you. We have added the number of the specimens for each molecular species

**Question:** Please clarify what is meant by “not including actual species”. For the first 3 genera (at least), the actual species seems to be listed many times (e.g. *Cx. quinquefasciatus, Ae. vexans*)

**Authors Answer:** “not including actual species” refers to result of morphological identification since there was possibility that the sample was incorrectly identified. We see the confusion this may create and have removed this. Thank you.

8. ***Question:*** *Lines 301-303: please clarify whether this “first-time evaluation..” applies globally, to Indonesia, to Sumba (or to some other level), to give a better understanding of the uniqueness of this rich dataset.*

**Authors Answer:** The data obtained from this study can be used as a reference for evaluation of the human biting mosquitoes in Indonesia. This study provides the first report to reveal the diversity of night biting, human host-seeking mosquito species not only for *Anophele*s, but also non-*Anopheles* species. We are not aware of other studies that have utilized non-*Anopheles* mosquitoes captured in malaria-centric studies – we prefer to be conservative and say this first time comprehensive evaluation applies to Indonesia.

**Minor comments**

1. ***Question*** *Line 52: I think “Anopheline” should not be capitalized. Please change to either Anophelinae or anopheline. (also line 54)*

**Authors Answer:** We have revised accordingly

2. ***Question****: Line 57: “Anopheles” should be italicized*

**Authors Answer:** We have revised accordingly

3. ***Question****: Lines 70-71: Please check the punctuation after “Culex”*

**Authors Answer:** We have revised accordingly

4. ***Question****: Line 89: the subfamilies typically end in -inae, so Toxorhynchites should be* *Toxorhynchitinae.*

**Authors Answer:** We have revised accordingly

5. ***Question****: Lines 108-110: I assume this is meant to say something like, “Characterizing species-specific traits…”.*

**Authors Answer:** We have restated this statement as suggested

6. ***Question****: Line 126: Is the ecological analysis mentioned here meant to be part of species identification per se? Or rather, is it that we can only accurately characterize species-specific ecology using data from accurately identified mosquito specimens?*

**Authors Answer:** We do agree with the reviewer that species-specific ecology may be relevant when the mosquito species is correctly identified.

7. ***Question****: Line 139 and Figure 2: should start date for rainfall data collection be June 2015? If not, where did data in Figure 2 come from prior to July 2016? Also, line 248 says, “Weather stations recorded rainfall and temperature throughout the study period”.*

**Authors Answer**: The weather station for the study was installed in July 2016. Data prior to this originated from a secondary weather station – but without rainfall. We have revised both the text and Figure 2.

8. ***Question****: Lines 140-142: please edit this section for clarity. Also, should this include temperature? (temperature is mentioned on line 180 but omitted in lines 140-142).*

**Authors Answer**: We have added into the sentence “rainfall and temperature” for clarity.

9. ***Question****: Figure 1: please add the number of houses at each cluster/star in the legend text.*

**Authors Answer:** The star figure in the map represents 1 coordinate sentinel house, there are 4 star figures in each cluster, but only one star visible due to the map scale and the distance between the sentinel houses.

10. ***Question****: Line 194: Should starting month be May 2015 as indicated in Abstract, Author Summary, and Methods? (Or else update the other 3 sections?)*

**Authors Answer:** We have corrected this. Thank you.

11. ***Question****: Line 220: Suggested change to “There were seven Aedes taxa identified molecularly”, or something other than species, given that five remained unidentified. Similar suggestion on lines 227 and 231.*

**Authors Answer:** Thank you. We have revised this accordingly and used ’taxa’ instead of ‘species’.

12. ***Question*** *Lines 253-254: in the text, correlation with rainfall is given for Culicinae, and stated as a positive correlation, but then the p-value is given as p<0.48, which would not be significant. Please check this section for accuracy.*

**Authors Answer:** Thank you. We have corrected this - no significant correlation was found between rainfall and temperature and the number of mosquitoes caught.

13. ***Question****: Table 2: some of the decimals are written as commas but should be changed to periods.*

**Authors Answer:** Thank you. We have revised this accordingly throughout the text.

14. ***Question****: Lines 254-255: “Peaks between rainfall highs were associated with agricultural irrigation.” Was this formally assessed in some way? If not, I would suggest to leave that for the discussion section.*

**Authors Answer**: We have moved this statement to the discussion section.

15. ***Question****: Lines 268-271: These lines could be removed given that the information is all contained in the figure.*

**Authors Answer:** Thank you. This has been revised accordingly

16. ***Question****: Lines 328-331: Clarify whether these species are known/documented JEV vectors.*

**Authors Answer:** Of the 4 Culex species identified, two species, *Cx. tritaenorhynchus* and *Cx. vishnui* have been documented to carry JEV (Garjito et al, 2018). We have added this to the manuscript.

17. ***Question****: Line 361: Clarify in this first sentence that the HBRs are specifically for evening/night, and not daytime biting.*

**Authors Answer:** we have clarified the statement accordingly that the HBR is specifically for evening/night only.

18. ***Question***: *References should be checked. For example:*
    a. *Ref. 3 is a study specifically about vectors of JEV in Indonesia, and so seems inappropriate as a citation for global mosquito taxa. A more appropriate reference would be, e.g.,* [*https://doi.org/10.1016/B978-012510451-7/50014-1*](https://doi.org/10.1016/B978-012510451-7/50014-1)
    b. *Ref. 6 is the World Malaria Report, but on line 96 is used as a reference for filarial worms and their vectors.*

**Authors Answer:** We have corrected the reference as suggested.

**General Comments;**

1. ***Question****: This work was initially mainly focused on malaria transmitting mosquitoes, but other species were also trapped during the study collection. Hence the findings might not be the true situation in the study location, for instance, Aedes spp. bites more during the day and may even bite throughout the day. Please indicate this a major limitation to this study. If possible, change the title to capture this development*

**Authors Answer:** Yes, we accept this limitation and have modified the title

accordingly. We want to emphasize that many non-*Anopheles* species are captured during malaria-centric studies and may be analysed – a benefit for our community.

2. ***Question****: The conclusion in the abstract needs to be rewritten to buttress on the key findings. The conclusion looks like justification for the study.*

**Authors Answer:** Thank you. We have revised the conclusion in the abstract

3. ***Question****: The methodology needs restructuring. It should be broken down into the appropriate sections. For example, a lot of things have been lumped together under the section study site; study area, study design and part of the statement for statistical or data analysis section*

**Authors Answer:** We have revised the methodology section according the reviewer has suggested. Methods have been subdivided into Study site and design, Mosquito collection, Mosquito identification, and an Analysis section.

4. ***Question****: The authors should avoid the indiscriminate use of hyphen as it makes most of the sentences not readable. Hyphen is better in separating words than sentences*

**Authors Answer:** Thank you. We have revised the relevant sentences suggested.

5. ***Question****: Hence the authors should see a statistician in order to improve the quality of the results and overall manuscript*

**Authors Answer:** We agree with the reviewer suggestion and have presented statistical analyses for all outputs.

6. ***Question****: If possible, the authors should try and dichotomize their mosquito data in a range, and run a mixed-effect-regression model to analyze the relationship between rainfall, temperature and abundance of different mosquito spp.*

**Authors Answer:** We do agree with the reviewer suggestion and conducted analysis accordingly.

7. ***Question****: No results were presented for monthly abundance alongside their entomological indices. You only presented general abundance.*

**Authors Answer**: This is correct. Monthly species-specific analysis for non-*Anopheles* was not possible due to the size of the sample analyzed (molecular and morphological identification) did not reflect overall populations.

8. ***Question****: Your figures are not clear*

**Authors Answer:** We have revised the figures

9. ***Question****: The discussion needs improvement as results were repeated and a lot of statements were not clear*

**Authors Answer**: We have revised the discussion as suggested.

10. ***Question****: It seems this study was carried out by inexperienced ‘entomologist’ as stated by the authors. Hence one major co-founders for large discrepancies recorded between morphological and molecular identification. Were they properly trained for the identification procedures before embarking on the study?*

**Authors Answer:** We acknowledges the reviewer point on the discrepancy between molecular and morphological identification. A large degree of the misidentification of non-*Anopheles* specimens is attributed to the lack of routine training of non-*Anopheles* mosquitoes (and a focus on *Anopheles*) – hence the requirement and added benefit of molecular methods. We agree that regular entomology training is required. The presence of cryptic species, novel species and species complexes, are also difficult to distinguish morphologically even by a well-trained entomologist.

**Other Comments:**

1. ***Question:*** *Line 89: Remove extra comma in Mansonia*

**Authors Answer:** We have corrected the relevant word accordingly.

2. ***Question:*** *Line 92: You can improve the readability of the statement by removing the hyphen and recast. E.g ………dry season owning to increased ………..*

**Authors Answer:** We have revised the sentences accordingly

3. ***Question:*** *Line 102: Sentence is too short, merge the two sentences together to make one statement*

**Authors Answer:** We have revised the sentences as suggested.

4. ***Question:*** Line 106: Remove the hyphen

**Authors Answer:** We have revised accordingly.

5. ***Question:*** Line 110-112: Statement is not clear, please recast.

**Authors Answer:** We have revised the sentence accordingly

6. ***Question*** *Line 130: Over multiple years? Be concise, (April 2016: April 2018)*

**Authors Answer:** We have revised the sentence as suggested

7. **Question** Line 142: Why spearman’s rho alone? Try and compute a linear regression model that will better quantify the magnitude of the relationships.

**Authors Answer:** We have tried to use both Spearman'rho and linear regression model as the reviewer suggested but both revealed no correlation.

8. ***Question****: Line 157 – 159: Your morphological identification followed which identification guide? Please recast line 157 – 159 as they need to be reconciled. Did you identify the whole specimen or you did a random identification? Or the random ones selected were to confirm what have been previously identified? Please clarify*

**Authors Answer:** Morphological species identification were carried out on all *Anopheles* using Rampa taxonomic keys. We have updated the text to reflect this. Non-*Anopheles* were identified to genera (since the study funding was focused on malaria vectors) with a subset (as updated in the methods) analyzed morphologically and molecularly. Briefly, Samples were stratified by morphological species (or genera for non-*Anopheles*), cluster, location, and time of capture, and a random subset (representing at least 10% of each morphologically identified taxa) spanning all strata were selected for identification (both morphological and molecular). For example, if there were 1000 specimens of a specific species/taxa identified, at least 10% (100 specimens) were randomly sampled to represent all sites, location of capture (inside and outside), and time of capture (across the night as well as over the course of the entire study).

9. ***Question****: Under your analysis section, there are statements that are meant to be written under study design or protocol. You shouldn’t be stating here how you collected your data. Just mention the method of analysis and their importance to the variables used in this study.*

**Authors Answer:** We have revised the relevant sentences accordingly.

10. ***Question****: The population of Aedes mosquitoes is low, could it be that it was under reported? Knowing well that it’s Aedes spp. can bite anytime of the day. However, I fully understood that this research was mainly focused on malaria transmitting mosquitoes according to your statements in some parts of your manuscript. It is therefore very important for you to properly state this a major limitation for your study.*

**Authors Answer:** This is correct. Since the collections were from 1800 h to 0600 h, many diurnal mosquitoes (Aedes) were not collected. We have recorded and clarified this throughout the manuscript to indicate the nocturnal nature of the data.

11. ***Question****: Line 199: Are the order listed for the Culex, Aedes and Amigeres species in ascending or descending orders with respect to abundance? It will be good if you can put their percentage abundance in a brackets in front of each species*

**Authors Answer:** We do agree with the reviewer suggestion and have revised the relevant sentences accordingly.

12. ***Question****:* Line 209: Recast as The remaining 8 ………………… representing ___% of the total *Anopheles* comprised of *An*…………..

**Authors Answer:** We have recasted the statement as suggested

13. ***Question****: Line 216 – 219: Remove and relocate to the appropriate section under methodology*

**Authors Answer:** we have revised accordingly

14. **Question**: Line 235-236: Please remove hyphen and recast. E.g. however, the observed similarity was below………….

**Authors Answer:** We have revised accordingly

15. **Question**: Line 201 – 252: Recast the statement and remove hyphen: e.g. Overall, there was an ------------Culicidae specimens (notably *Culex* and *Anopheles*) during the raining season

**Authors Answer:** We have revised the sentence. Thank you.

16. ***Question****: Line 254: P<0.48 is not correct for significant relationship. Rather put (r values ranging between 0.485 – 0.552, p<0.05).*

**Authors Answer:** We have corrected this point accordingly. The r values 0.3 and 0.13 represent temperature and rainfall, respectively.

17. ***Question****: How did you determine your human biting rates? This wasn’t spelt out in the methodology section*

**Authors Answer:** We have updated this in the methods. HBR was calculated as bites per person per night or bites per person per hour.

18. ***Question****: Line 322: fewer studies and lack of morphological identification experience? Do you have any literature to back it up? Are you saying the identifiers were not properly trained for morphological identification?*

**Authors Answer**: We have updated the discussion to reflect that there might have been multiple factors that contributed to this misidentification – especially in non-*Anopheles* specimens. This may be attributed to a historically lesser focus on the development and validation of non-*Anopheles* morphological keys combined less research and consequently less experience on morphological identification related to these species. The presence of species complexes, sibling species and novel species in baseline characterizations (such as this) further complicates morphological identification.

19. ***Question****: Line 327: Data that is supported? Statement not clear, please recast*

**Authors Answer:** We have recast the sentence as suggested

20. *Line 373: Remove figure 5. Also avoid stating your results in the discussion section*

**Authors Answer:** We have corrected the relevant sentences as suggested
